# Increased risk of laryngeal and pharyngeal cancer after gastrectomy for ulcer disease in a population-based cohort study

**DOI:** 10.1038/bjc.2012.72

**Published:** 2012-03-08

**Authors:** J Lagergren, A Lindam

**Affiliations:** 1 Department of Molecular Medicine and Surgery, Upper Gastrointestinal Research, Karolinska Institutet, Norra Stationsgatan 67, Level 2, 17176 Stockholm, Sweden; 2 Division of Cancer Studies, King's College London, London, UK

**Keywords:** gastric surgery, gastric resection, bile reflux, duodeno-gastro-oesophageal reflux, oesophageal cancer

## Abstract

**Background:**

Gastrectomy has been indicated as a risk factor for laryngeal cancer, and possibly also for pharyngeal cancer, but few studies are available. The postulated mechanism is increased bile reflux following gastrectomy.

**Methods:**

This was a population-based cohort study of patients who underwent gastrectomy for peptic ulcer disease between 1964 and 2008 in Sweden. Follow-up data for cancer was obtained from the Swedish Cancer Register. Relative risk was calculated as standardised incidence ratios (SIRs) with 95% confidence intervals (CIs).

**Results:**

The gastrectomy cohort comprises 19 767 patients, contributing 348 231 person-years at risk. The observed number of patients with laryngeal (*n*=56) and pharyngeal cancer (*n*=28) was two-fold higher than the expected (SIR: 2.0, 95% CI: 1.5–2.6 and SIR: 2.4, 95% CI: 1.6–3.5, respectively). After exclusion of 5536 cohort members with tobacco- or alcohol-related disease, the point SIRs remained increased (SIR: 1.6, 95% CI: 1.1–2.2 and SIR: 1.7, 95% CI: 0.9–2.8, respectively). The SIRs of laryngeal and pharyngeal cancer increased with time after gastrectomy (*P* for trend <0.0001), and were particularly increased ⩾30 years after gastrectomy (SIR: 4.8, 95% CI: 2.1–9.5 and SIR: 10.2, 95% CI: 3.7–22.3, respectively).

**Conclusion:**

Gastrectomy for peptic ulcer disease might entail a long-term increased risk of laryngeal and pharyngeal cancer.

The only established risk factors for laryngeal and pharyngeal squamous cell carcinoma are high consumption of tobacco and alcohol (Zeka *et al*, 2003). An association between oesophageal biliary regurgitation and these tumours has also been indicated, particularly laryngeal cancer, but this relation is debated (Galli *et al*, 2006a, 2006b). The lack of protective mechanisms against proximal reflux has been proposed to contribute to the harmful effect, but any influence of bile regurgitation on the development of laryngeal and pharyngeal cancer is difficult to assess in humans. Problems arise from difficulties with assessing exposure to bile reflux, and the long expected latency period before invasive tumours will develop. The specific situation occurring after partial or total gastrectomy, however, resembles a human experimental model of bile reflux, since the anatomical rearrangement after such surgery facilitates the flow of duodenal contents to reach the oesophagus. A large proportion of patients who have undergone gastrectomy suffer from oesophageal bile regurgitation (Stoker and Williams, 1991; Matei *et al*, 2010). Thus, if bile regurgitation is a carcinogenic exposure for the larynx or pharynx, gastrectomy could be followed by an increased risk of tumours at these sites. This hypothesis has gained support from a limited number of investigations, including a large case–control study showing a four-fold increased risk of laryngeal cancer among patients who reported having undergone a gastrectomy (Cammarota *et al*, 2004). However, no cohort studies are available. To further evaluate the relation between bile regurgitation and risk of laryngeal and pharyngeal cancer, we conducted a large cohort study assessing the association between gastrectomy for peptic ulcer disease and risk of developing these tumours.

## Materials and Methods

### Design

This was a Swedish population-based cohort study addressing the risk of developing laryngeal or pharyngeal cancer after gastrectomy for peptic ulcer disease, using the entire Swedish population during the period of 1964–2008 as a database. The nationwide Swedish Patient Register was used to identify a cohort of all patients who had undergone gastrectomy for peptic ulcer disease during the study period in Sweden. New tumours in the gastrectomy cohort were identified through linkage to the nationwide Swedish Cancer Register. The incidence of laryngeal or pharyngeal cancer among the gastrectomy patients was compared with the incidence in the Swedish background population of the corresponding age, sex, and calendar year. The cancers occurring in the comparison population were assessed through the data on the cancer incidence and prevalence in the Swedish Cancer Register. Both in the study cohort and in the comparison population, only first tumours were included. In addition, the first year after gastrectomy was excluded to reduce the risk of earlier detection of any cancer because of the gastrectomy, and to allow a minimum time of exposure. To obtain the correct censoring of person-time, the dates of all deaths were collected through linkage to the Swedish Cause of Death Register. The personal identity number, a 10-digit number assigned to every resident in Sweden since 1947, made the linkage of individuals across registers possible (Ludvigsson *et al*, 2009). The study was approved by the Regional Ethics Committee in Stockholm.

### Gastrectomy cohort

Patients who underwent gastrectomy for peptic ulcer disease were identified through the Swedish Patient Register, containing data on all hospitalisations and surgical procedures performed in Sweden since 1964. The Swedish Patient Register, administered by the National Board of Health and Welfare, includes data on the patients’ age, sex, personal identity number, discharge diagnoses and surgical procedures, and the dates of each hospitalisation. Sixty percent of the Swedish population was covered by this register in 1969 and 85% in 1983, and since 1987 the coverage has been 100%. Validation studies of the operation codes in this register have reported 99% completeness and 95% correctness (Falkeborn *et al*, 1995). The diagnosis codes representing peptic ulcer disease were defined by the International Classification of Diseases (ICD) (version 7: 540, 541, 542, 543, 544, and 545; version 8: 531, 532, 533, 534, 535, 536, and 537; version 9: 531, 532, 533, 534, 535, 536, and 537; and version 10: K25, K26, K27, K28, K29, and K31). Among the peptic ulcer patients, the gastrectomy cohort included the patients who had undergone partial or total gastrectomy according to the Patient Register, with operation codes defined by the Swedish Classification of Surgical Procedures edition 5 (4421, 4423, 4439; edition 6: 4411, 4412, 4413, 4414, 4415, 4416, 4417, 4418, 4419, 4420, 4422, 4425, 4426, 4429, 4430, 4432, 4434, and 4435; and edition 7: JDC and JDD).

### Detection of laryngeal and pharyngeal cancer

All cancers diagnosed during follow-up of the cohort (1965–2008) were identified through linkage to the Swedish Cancer Register, a nationwide register initiated in 1958 and administered by the National Board of Health and Welfare, which is at least 98% complete (Barlow *et al*, 2009). The register contains information about the location, histological type, and date of diagnosis of all malignant tumours in Sweden. The codes for all tumour diagnoses are converted into the ICD codes version 7, and the study outcomes were defined by the codes 161 for laryngeal cancer and 146–148 for pharyngeal cancer, combined with the histological code 146 for squamous cell carcinoma.

### Statistical analyses

Person-time at risk was accumulated from 1 year after the surgery until the first occurrence of any cancer, death, or the end of observation (31 December 2008), whichever came first. The relative risk was estimated as the standardised incidence ratio (SIR), that is, the observed number of laryngeal or pharyngeal tumours in the gastrectomy cohort divided by the number of these tumours in the comparison population (expected number). The expected number of cancers was calculated by multiplying the observed person-time by cancer incidence rates specific for age, sex, and calendar year. The expected rates were derived from the Swedish Cancer Register data through the Swedish population, and aggregated into 5-year intervals. The SIRs were inherently adjusted for the potential confounding factors age, sex, and calendar year, as the incidence in the observed cohort was compared with the corresponding incidence in the age-, sex-, and calendar year-matched general population. Confidence intervals (CIs) of SIRs were calculated on the assumption that the observed number of events followed a Poisson distribution (Breslow and Day, 1987).

To evaluate confounding by tobacco smoking and overconsumption of alcohol, sensitivity analyses were conducted, excluding all individuals and person-years with a diagnosis representing alcohol overconsumption or tobacco smoking in the inpatient or outpatient care, as recorded in the Swedish Patient Register. Alcohol-related diagnoses included a history of excessive alcohol consumption (diagnosis code F10 in ICD-10, 291 or 303 in ICD-9 and ICD-8, or 307 or 322 in ICD-7) or vitamin B deficiency associated with alcohol (E51–52 or G62.1 in ICD-10, 265 in ICD-9, 261.00–262.00 in ICD-8, or 280–281 in ICD-7) or liver disease related to alcohol intake (K70 in ICD-10, 571.A or 571.C in ICD-9, 571.00 or 571.01 in ICD-8, 581.10 or 583.10 in ICD-7). Tobacco smoking was assessed by smoking-related diseases, including chronic obstructive pulmonary disease or bronchitis (J41–J44 in ICD-10, 490–492 in ICD-9 and ICD-8, 501.99, 502, 527.10, or 527.11 in ICD-7), or atherosclerosis or peripheral vascular disease (I70 or I73.9 in ICD-10, 440 or 443.x in ICD-9, 440, 443.90 or 445 in ICD-8, or 450.00, 450.10, or 453.33 in ICD-7).

The Statistical Analysis System (SAS), version 9.2, SAS Institute Inc., Gary, NC, USA, was used for all analyses.

## Results

### Patients

The study cohort included 19 767 patients who had undergone gastrectomy. Some characteristics of these patients are presented in Table 1. The follow-up time was a median of 17 years, providing 348 231 person-years at risk. There was a male predominance (62.3%). Most operations were partial gastrectomies (96.4%), while the remaining proportion represented total gastrectomies.

### Risk of laryngeal cancer

A total of 56 patients developed laryngeal cancer at least 1 year after the gastrectomy, rendering a two-fold higher risk than expected (SIR: 2.0, 95% CI: 1.5–2.6) (Table 2). The risk was further increased in periods representing longer latency intervals after gastrectomy, that is, longer time of exposure (*P* for trend <0.0001). Among those who were operated on at least 20 years earlier, the SIR was 2.7 (95% CI: 1.6–4.2), and among those who were operated on at least 30 years earlier, the SIR was nearly a five-fold increase (SIR: 4.8, 95% CI: 2.1–9.5). There were no substantial differences between sexes, age groups, or calendar periods regarding risk of laryngeal cancer after the gastrectomy (Table 2). Analyses restricted to patients with a histologically confirmed squamous cell carcinoma of the larynx revealed similar results (data not shown). After exclusion of 5536 cohort members with any disease linked with tobacco smoking or alcohol abuse, the overall SIR remained increased (SIR: 1.6, 95% CI: 1.1–2.2). The SIR was 2.2 (95% CI: 1.1–4.0) at least 20 years after the gastrectomy, and the SIR was 4.0 (95% CI: 1.3–9.3) among patients who were followed up for at least 30 years (Table 3).

### Risk of pharyngeal cancer

A total of 28 patients developed pharyngeal cancer during the follow-up of the gastrectomy cohort. The overall risk of pharyngeal cancer was over two-fold higher than expected (SIR: 2.4, 95% CI: 1.6–3.5). The SIR was particularly increased in periods representing longer exposure time after gastrectomy. Among patients who were operated on at least 30 years earlier, there was a 10-fold increased risk (SIR: 10.2, 95% CI: 3.7–22.3). There were no notable differences between sexes or age groups after the gastrectomy, while the SIR was higher in the later calendar period (Table 2). The results based only on patients with a histologically confirmed squamous cell carcinoma of the pharynx were similar (data not shown). After exclusion of cohort members with any disease linked with tobacco smoking or alcohol abuse, the SIRs decreased (Table 3). The overall SIR was 1.7 (95% CI: 0.9–2.8), and after at least 30 years of follow-up the SIR was 2.3 (95% CI: 0.5–16.3).

## Discussion

This study supports the hypothesis that gastrectomy increases the risk of laryngeal and pharyngeal cancer.

Methodological advantages of the study include the population-based cohort design, the complete assessment of the exposure and the outcomes, the long and complete follow-up, and the large sample size. The use of a gastrectomy cohort mimics an experimental model that allows the assessment of the effects of the exposure to bile reflux in humans. Adjustments for age, sex, and calendar year were achieved through the design, but a drawback was the lack of direct data on tobacco smoking and alcohol abuse, the established risk factors for these tumours (Zeka *et al*, 2003). Nevertheless, it was possible to address confounding by these factors by excluding all patients who had ever been recorded with a diagnosis linked with tobacco smoking or high alcohol consumption in the Patient Register.

The results of the present study are in line with those reported in a limited number of studies that have addressed the relation between gastrectomy and risk of laryngeal and pharyngeal cancer. The hypothesis of an association between gastrectomy and laryngeal or pharyngeal cancer has been put forward in a small case series that demonstrated abnormal bile reflux in three patients with laryngeal cancer after gastric resection (Cianci *et al*, 2000). This finding was followed up by a case–control study of 40 gastrectomy patients with biliary reflux due to gastrectomy and 40 non-gastrectomy dyspeptic patients, which provided some further support for the hypothesis that gastrectomy is a risk factor for precancerous and squamous cell carcinoma of the larynx or pharynx (Galli *et al*, 2002). Another case–control study compared the presence of laryngeal lesions in 93 patients who had undergone gastric resection with 93 matched dyspeptic controls, and found that seven patients (7.5%) had malignant or premalignant laryngeal lesions in the gastrectomy group, while one control patient had a lesion. Finally, a large case–control study found that previous gastrectomy was reported by 8.1% of the 828 cases of laryngeal cancer and by 1.8% of the 825 matched controls with myocardial infarction, and the relative risk was 3.8 (95% CI: 2.1–7.0) after adjustment for tobacco smoking and alcohol consumption (Cammarota *et al*, 2004). The association was strongly dependent on latency time; the odds ratio was 14.8 (95% CI: 3.4–64.6) ⩾20 years after the gastrectomy. The finding of a stronger association with longer follow-up is in agreement with the findings of the present study. Taken together, although based on a scarce number of studies, there are data to support an association between gastrectomy and risk of laryngeal and pharyngeal cancer.

The biological mechanism behind a possible association between gastrectomy and these tumours is uncertain. There have been a number of studies performed and some have provided evidence that acid reflux may be a risk factor for laryngeal cancer (Qadeer *et al*, 2005). The situation that occurs after partial or total gastrectomy mimics a human experimental model of bile reflux, since such surgery is often followed by a substantially increased risk of oesophageal exposure to bile (Toye and Williams, 1965; Yumiba *et al*, 2002; Matei *et al*, 2010). The anatomical rearrangement after gastrectomy means that duodenal contents easily flows back and can reach the oesophagus, the pharynx, and the larynx. The pharynx and larynx have no defence mechanisms against such proximal reflux, for example, no peristalsis, and such reflux might cause chronic injury and inflammation on the epithelium (Glanz and Kleinsasser, 1976). Tobacco smoking and alcohol consumption, the main risk factors for laryngeal and pharyngeal cancer, might increase the vulnerability of the mucosa for such reflux exposure.

In conclusion, this large and population-based cohort study with long and complete follow-up provides further support for the hypothesis that gastrectomy increases the risk of squamous cell carcinoma of the larynx and pharynx.

## Figures and Tables

**Table 1:** Characteristics of the 19 767 study patients who underwent gastrectomy for peptic ulcer disease in Sweden during the period 1964–2008

**Variable**	**Men**	**Women**	**All**
Individuals, number (%)	12 391 (62.7)	7376 (37.3)	19 767
Person-years, number (%)	213 846 (63.9)	120 773 (36.1)	334 619
Median age at entry, years	57	60	58
Median time at entry, calendar year	1976	1980	1978
Median follow-up time, years	17	16	17
			
*Type of surgery, number (%)*			
Partial gastrectomy	11 986 (96.7)	7061 (95.7)	19 047 (96.4)
Total gastrectomy	405 (3.3)	315 (4.3)	720 (3.6)

**Table 2:** Standard incidence ratios (SIRs) and 95% confidence intervals (CIs) of laryngeal and pharyngeal cancer in a gastric resection cohort, consisting of 19 767 patients in Sweden during the period 1964–2008

	**Laryngeal cancer**			**Pharyngeal cancer**		
**Variable**	**Observed cases (*n*)**	**Expected cases (*n*)**	**SIR (95% CI)**	**Observed cases (*n*)**	**Expected cases (*n*)**	**SIR (95% CI)**
All	56	28.5	2.0 (1.5–2.6)	28	11.7	2.4 (1.6–3.5)
						
*Gender*						
Male	50	26.6	1.9 (1.4–2.5)	25	10.0	2.5 (1.6–3.7)
Female	6	1.9	3.1 (1.1–6.8)	3	1.7	1.7 (0.4–3.3)
						
*Age (years)*						
<70	36	14.3	2.5 (1.8–3.5)	13	5.9	2.2 (1.2–3.8)
⩾70	20	14.2	1.4 (0.9–2.2)	15	5.8	2.6 (1.4–4.3)
						
*Latency (years)*						
1–10	17	12.2	1.4 (0.8–2.2)	11	5.3	2.1 (1.0–3.7)
11–20	21	9.6	2.2 (1.4–3.4)	8	3.9	2.0 (0.9–4.0)
21–30	10	5.1	2.0 (0.9–3.6)	3	1.9	1.6 (0.3–4.5)
⩾30	8	1.7	4.8 (2.1–9.5)	6	0.6	10.2 (3.7–22.3)
						
*Calendar period*						
1965–1988	24	14.3	1.8 (1.1–2.5)	10	6.2	1.6 (0.8–3.0)
1989–1998	19	8.7	2.2 (1.3–3.4)	8	3.5	2.3 (1.0–4.5)
1999–2008	13	5.6	2.3 (1.2–4.0)	10	2.1	4.9 (2.3–8.9)

**Table 3:** Standard incidence ratios (SIRs) and 95% confidence intervals (CIs) of laryngeal and pharyngeal cancer in a gastric resection cohort, consisting of 14 231 patients in Sweden during the period 1964–2008 after exclusion of 5536 who had any recorded diagnosis of tobacco-related or alcohol-related disease (chronic obstructive pulmonary disease or bronchitis, atherosclerosis, peripheral vascular disease, excessive alcohol consumption, vitamin B deficiency associated with alcohol, and liver disease related to alcohol intake)

	**Laryngeal cancer**			**Pharyngeal cancer**		
**Variable**	**Observed cases (*n*)**	**Expected cases (*n*)**	**SIR (95% CI)**	**Observed cases (*n*)**	**Expected cases (*n*)**	**SIR (95% CI)**
All	32	20.5	1.6 (1.1–2.2)	14	8.5	1.7 (0.9–2.8)
						
*Gender*						
Male	31	19.1	1.6 (1.1–2.3)	12	7.1	1.7 (0.9–2.9)
Female	1	1.4	0.7 (0.0–3.9)	2	1.3	1.5 (0.2–5.4)
						
*Age (years)*						
<70	18	9.9	1.8 (1.1–2.9)	8	4.1	2.0 (0.8–3.8)
⩾70	14	10.6	1.3 (0.7–2.2)	6	4.4	1.4 (0.5–3.0)
						
*Latency (years)*						
1–10	8	8.8	0.9 (0.4–1.8)	7	3.8	1.8 (0.7–3.8)
11–20	13	6.8	1.9 (1.0–3.3)	3	2.8	1.1 (0.2–3.1)
21–30	6	3.7	1.6 (0.6–3.3)	2	1.4	1.4 (0.2–5.2)
⩾30	5	1.3	4.0 (1.3–9.3)	2	4.5	2.3 (0.5–16.3)
						
*Calendar period*						
1965–1988	16	10.2	1.6 (0.9–2.5)	5	4.5	1.1 (0.4–2.6)
1989–1998	9	6.2	1.5 (0.7–2.8)	5	2.5	2.0 (0.6–4.7)
1999–2008	7	4.1	1.7 (0.7–3.5)	4	1.5	2.6 (0.7–6.8)

## References

[bib1] Barlow L, Westergren K, Holmberg L, Talback M (2009) The completeness of the Swedish Cancer Register: a sample survey for year 1998. Acta Oncol 48: 27–33 18767000 10.1080/02841860802247664

[bib2] Breslow NE, Day NE (1987) The Design and Analysis of Cohort Studies. IARC Scientific Publications no 82, International Agency for Research on Cancer: Lyon 3329634

[bib3] Cammarota G, Galli J, Cianci R, De Corso E, Pasceri V, Palli D, Masala G, Buffon A, Gasbarrini A, Almadori G, Paludetti G, Gasbarrini G, Maurizi M (2004) Association of laryngeal cancer with previous gastric resection. Ann Surg 240: 817–824 15492563 10.1097/01.sla.0000143244.76135.ca PMC1356487

[bib4] Cianci R, Fedeli G, Cammarota G, Galli J, Agostino S, Di Girolamo S, Maurizi M, Gasbarrini G (2000) Is the risk alkaline reflux a risk factor for laryngeal lesions? Am J Gastroenterol 95: 2398 11007261 10.1111/j.1572-0241.2000.02352.x

[bib5] Falkeborn M, Persson I, Naessen T, Kressner U (1995) Validity of information on gynecological operations in the Swedish in-patient registry. Scand J Soc Med 23: 220–224 8602494 10.1177/140349489502300314

[bib6] Galli J, Cammarota G, Calo L, Agostino S, D’Ugo D, Cianci R, Almadori G (2002) The role of acid and alkaline reflux in laryngeal squamous cell carcinoma. Laryngoscope 112: 1861–1865 12368631 10.1097/00005537-200210000-00030

[bib7] Galli J, Cammarota G, De Corso E, Agostino S, Cianci R, Almadori G, Paludetti G (2006a) Biliary laryngopharyngeal reflux: a new pathological entity. Curr Opin Otolaryngol Head Neck Surg 14: 128–132 16728887 10.1097/01.moo.0000193198.40096.be

[bib8] Galli J, Cammarota G, Volante M, De Corso E, Almadori G, Paludetti G (2006b) Laryngeal carcinoma and laryngo-pharyngeal reflux disease. Acta Otorhinolaryngol Ital 26: 260–263 17345929 PMC2639966

[bib9] Glanz H, Kleinsasser O (1976) Chronic laryngitis and carcinoma (author's transl). Arch Otorhinolaryngol 212: 57–75 989307 10.1007/BF00456363

[bib10] Ludvigsson JF, Otterblad-Olausson P, Pettersson BU, Ekbom A (2009) The Swedish personal identity number: possibilities and pitfalls in healthcare and medical research. Eur J Epidemiol 24: 659–667 19504049 10.1007/s10654-009-9350-y PMC2773709

[bib11] Matei D, Dadu R, Prundus R, Danci I, Ciobanu L, Mocan T, Bocsan C, Zaharie R, Serban A, Tantau M, Iancu C, Alexandru I, Al-Hajjar N, Andreica V (2010) Alkaline reflux esophagitis in patients with total gastrectomy and Roux en Y esojejunostomy. J Gastrointestin Liver Dis 19: 247–252 20922186

[bib12] Qadeer MA, Colabianchi N, Vaezi MF (2005) Is GERD a risk factor for laryngeal cancer? Laryngoscope 115: 486–491 15744163 10.1097/01.mlg.0000157851.24272.41

[bib13] Stoker DL, Williams JG (1991) Alkaline reflux oesophagitis. Gut 32: 1090–1092 1955159 10.1136/gut.32.10.1090 PMC1379364

[bib14] Toye DK, Williams JA (1965) Post-gastrectomy bile vomiting. Lancet 2: 524–526 4158177 10.1016/s0140-6736(65)91475-3

[bib15] Yumiba T, Kawahara H, Nishikawa K, Inoue Y, Ito T, Matsuda H (2002) Impact of esophageal bile exposure on the genesis of reflux esophagitis in the absence of gastric acid after total gastrectomy. Am J Gastroenterol 97: 1647–1652 12135013 10.1111/j.1572-0241.2002.05822.x

[bib16] Zeka A, Gore R, Kriebel D (2003) Effects of alcohol and tobacco on aerodigestive cancer risks: a meta-regression analysis. Cancer Causes Control 14: 897–906 14682447 10.1023/b:caco.0000003854.34221.a8

